# Associations between breastfeeding and self-reported experience of the “10 Steps to Successful Breastfeeding”: a cross-sectional and longitudinal study of maternity clinic practices in Cyprus

**DOI:** 10.3389/fgwh.2024.1420670

**Published:** 2024-12-16

**Authors:** Mary Economou, Ourania Kolokotroni, Irene Paphiti-Demetriou, Christiana Kouta, Ekaterini Lambrinou, Eleni Hadjigeorgiou, Vasiliki Hadjiona, Nicos Middleton

**Affiliations:** ^1^ Department of Nursing, School of Health Sciences, Cyprus University of Technology, Limassol, Cyprus; ^2^ Cyprus Breastfeeding Association – ‘Gift for Life’, Nicosia, Cyprus

**Keywords:** Breastfeeding, 10 Steps to Successful Breastfeeding, maternity practices, skin-to-skin or pacifier, exclusivity

## Abstract

**Objective:**

To assess the implementation of the “10 Steps for Successful Breastfeeding” and explore associations with any Breastfeeding (BF) and Exclusive Breastfeeding (EBF) initiation and continuation.

**Methods:**

Implementation of the 10 Steps was assessed based on a consecutive sample of 568 mothers’ self-reported experience across all public (*N* = 5) and 29 (of 35) private maternity clinics using the WHO/UNICEF BFHI questionnaire (Section 4) within the first 24–48 h after birth. BF and EBF were estimated within 48 h after birth as well as at 1st, 4th and 6th month based on a self-reported current status method. Associations with initiation and continuation of any BF and EBF up to the 6th month were explored in logistic regression after adjusting for potential confounders. Associations were explored with individual steps as well as the overall experience, operationalized as the sum score of full, partial or no implementation of each item, with the exception of Step 6 (exclusive breastfeeding).

**Results:**

At mean score 6.2 (SD = 2.7), the overall 10 Steps experience was low (theoretical range 0–14), even among those who breastfed exclusively (M = 7.7, SD = 2.0). EBF and BF initiation and continuation showed a stepwise association with self-reported experience of the 10 Steps. Across quartiles of increasing scores within 48 h after birth, the prevalence of EBF was 7.5%, 14.4%, 19.0%, and 34.2%. Mothers who experienced full or partial implementation of Step 4 (i.e., early initiation with skin-to-skin) were more likely to continue BF and EBF up to the 4th month, while use of pacifiers (Step 9) was more likely to have an adverse effect on breastfeeding continuation and exclusivity.

**Conclusion:**

The BFIH's “10 Steps” are associated with BF initiation while certain practices seem to be more strongly associated with exclusivity and continuation of breastfeeding up to the sixth month. While breastfeeding intention may determine the experience of the 10 Steps, this also suggests that maternity care practices can have a supportive role for mothers to succeed their intentions to BF and EBF.

## Introduction

Exclusive Breastfeeding is the optimal source of nourishment for infants during the first six months, imparting immediate along with enduring life-lasting positive effects (1–3). The “*10 Steps for Successful Breastfeeding*’’ delineate crucial maternity healthcare practices aimed at bolstering maternal breastfeeding initiation. Since the launch of the *Baby Friendly Hospital Initiative* (BFHI) in 1991, a global initiative established by WHO and UNICEF, the “*Ten Steps to Successful Breastfeeding*” (4) has served as a fundamental component of both national and international strategies for several decades. The BFHI aims to enhance maternity care services by emphasizing the adherence of maternity care facilities to good practices which support and safeguard breastfeeding. Initially, the development of the “10 Steps” relied on clinical experience rather than research evidence regarding their impact in influencing breastfeeding behavior and outcomes (4). However, numerous studies since then, utilizing both observational and intervention study designs, have consistently demonstrated that adherence to the “10 Steps” is associated with higher rates of breastfeeding initiation and longer breastfeeding duration. (5–8).

Nevertheless, suboptimal implementation the “10 Steps” has been documented in many countries worldwide (9–14). In commemorating the 25th Anniversary of the initiative's launch, the WHO (2017) published an analysis of the current status of the BFHI worldwide. The overall estimate indicated that approximately 10% of births took place in hospitals designated as Baby-Friendly, with variations from less than 5% in Africa and Southeast Asia to 36% in the European region. Furthermore, notable disparities were noted between countries, even within the European region. Only twelve out of 47 countries in the WHO European region reported that over 50% of births occurred in baby-friendly designated hospitals, while 16 countries reported having no designated facilities (15).

In the absence of a national BFHI accreditation programme in Cyprus, there are no Baby Friendly maternity hospitals or clinics. Even though research findings suggest that initiation of breastfeeding appears high at 84% within the first 48 h after birth, only 1 in 5 mothers initiate exclusive breastfeeding (16). The rate of exclusive breastfeeding drops considerably after the first month and, by the sixth month, only 5% of new mothers are exclusively breastfeeding, falling short of the WHO target of at least 50% of infants aged under six months being exclusively breastfed (17). Despite the fact that the National Breastfeeding Committee of the Ministry of Health in Cyprus released a National Strategy (2011) and a National Policy (2015) for breastfeeding, largely focusing on promoting the implementation of the “10 Steps”, breastfeeding rates remain low, while maternity clinics’ practices are neither standardized nor monitored. This considerable heterogeneity in practices across maternity clinics in Cyprus causes maternal insecurity and confusion with an increased chance of failure to breastfeed (18). In recent years, the Cyprus National Committee for Breastfeeding has embarked on developing the first BFHI accreditation of maternity clinics, which was nevertheless stalled due to the COVID-19 pandemic.

In addition to several anecdotal reports of suboptimal practices, there is evidence of fragmented implementation of the “Ten Steps” in a sample of maternity units in the capital city Nicosia, as part of the “BrEaST start in life” program (19). The study showed that the highest degree of implementation was for Step 5 (practical assistance by the healthcare staff). In contrast, almost none of the mothers experienced Step 9 (no pacifiers or soothers to infants), whilst there appeared to be poor implementation of Step 7 (rooming-in) and Step 10 (community support after discharge). In the private sector, practices such as rooming-in and skin-to-skin are primarily implemented at the request of the mother. Hospitals in the public sector are more likely to implement the “10 Steps” for all mothers who choose to breastfeed. Breastfeeding support is often limited during the mother-infant dyad's stay in the maternity clinic, which typically lasts about 2–3 days. Support clinics are rare and if mothers desire additional support beyond what the maternity clinic provides, they may seek support from a lactation consultant at their own initiative and expense. Beyond that, community support is restricted to services provided by NGOs, such as the Cyprus Breastfeeding Association “Gift for life” which offer free monthly group meetings and a telephone support line. An important development after the recent re-structuring of the General Healthcare System (GeSY) is the introduction of community midwifery. Direct access to a midwife will be reimbursed by the system for up to a total of six visits, with quotas depending whether these visits are performed prenatally or antenatally. This service was not in place at the time of the study, and it is too early to evaluate the impact it will have on national breastfeeding rates.

In view of the first BFHI accreditation scheme in the country, the aim of this study was to quantify the association of the maternal self-reported implementation of the “10 Steps” with initiation, exclusivity and continuation of breastfeeding among women giving birth across a nationwide sample of maternity clinics in Cyprus.

## Methods

### Study design

A cross-sectional and longitudinal observational study, conducted between April 2014 and June 2015,. The study was part of the wider research program “BrEaST start in life: *addressing social inequalities and supporting breastfeeding through inclusion activities*”. A consecutive nationwide sample of mother-infant dyads was recruited during their stay at maternity clinics over a period of 6–8 weeks from all public hospitals (5 in total) and 29 of 35 private clinics. At baseline, mothers were provided with the opportunity to participate in the first phase of the study (maternal assessment of the implementation of the 10 Steps), even if they did not wish to participate in the follow-up phases (continuation and exclusivity of breastfeeding). Mothers were asked to respond to a set of questionnairess that included the battery of tools described below. Mothers who provided contact details (phone and/or email) were followed-up prospectively with a telephone interview at the first, fourth and six months postpartum. The study design, was described in detail in previous publications (16, 20). As previously reported, no significant differences in terms of socio-demographic characteristics, intention to breastfeed or breastfeeding self-efficacy were observed between women who participated in the follow-up phases and those who participated at baseline only.

### Sample size

We conducted precision analysis with finite population correction (as annual number of births is around 10,000). According to the analysis, the minimum required sample size to estimate the prevalence of breastfeeding with 95% confidence interval not wider that ±5% was 370 participants. The potential non-participation in the follow-up was accounted for by inflating the sample size recruited at baseline Information about the calculation of the sample size was described in previous publications (16, 20).

### Eligibility criteria

Mothers who participated in the study met the following criteria: 1. they gave birth to a live infant, 3. they were at least 18 years old, 4. they could read or speak Greek or English, 5. they had no health conditions that would prevent breastfeeding (e.g., bilateral mastectomy, postpartum maternal complications) and 6. they were not separated from their infants after birth for medical reason that would prevent breastfeeding initiation within one hour after birth (e.g., transferred to NICU at the same hospital or at a different location).

### Data collection and tools

#### Estimation of Bf and EBF indicators

At baseline, mothers completed a questionnaire pack which included the self-administered WHO UNICEF questionnaire - Section 4 (21). BF and EBF were estimated based on the WHO definitions (22). Exclusive breastfeeding was defined as the infant receiving solely breast milk, with the exception of medications, vitamin or mineral supplements, or oral rehydration solutions. An infant was considered to be breastfeeding if they received breast milk in addition to any other liquids or foods, including formula.

BF initiation was derived from the response to the question “Have you breastfed your newborn baby in the first 48 h?”, included in the baseline questionnaire. To estimate exclusivity in BF, the set of questions referring to Step 7 “Implementation of Exclusive Breastfeeding”, as phrased in Section 4 of the BFHI (Baby Friendly Hospital Initiative) questionnaire for mothers’ self-assessment of maternity unit practices were used (21). This was based on negative responses to the following questions (1) “*Did your baby receive any supplement*?’’ and (2) “*Did your baby receive any of the following?”* referring to any supplementation (formula, water or sugar water, other fluids).

At the first month of follow-up, mothers were asked about the type, timing and frequency of supplemental feeding including formula, other liquids, solids, medication, vitamin or mineral drops and Oral Rehydration Solution (ORS) using both the self-reported current status and the 24hr recall method. By the fourth and sixth month, additional information was gathered regarding the introduction, type and frequency of solids. Infant feeding practices at fourth and sixth month were assessed based on self-reported current status, a 24-hour recall as well as a retrospective event calendar method, which included the following questions: “ *Until today has your baby ever had (a) Fruit cream/puree (b) Other creams (e.g farin lactee, rice cream) and (c) Mashed or solid food?’’* and “*How often does your baby have (a) fruit cream/puree, (b) other creams (e.g farin lactee, rice cream) and (c) Mashed or solid food*?’’.

#### Assessment of the implementation of ten steps

The WHO/UNICEF Baby-Friendly Hospital Initiative package (Section 4) for self-assessment and monitoring of Baby Friendly Hospitals was used. This tool was directed to mothers while at the clinic after birth. “*The 10 Steps for Successful Breastfeeding*” self-assessment and monitoring questionnaire was developed to gather feedback from mothers about their experiences of BFHI's “*10 Steps*”. This practical tool allows maternity clinics to evaluate their adherence to recommended maternity practices (21) and forms part of an accreditation process. It covers Steps 3 through 10 and the International Code for Breastfeeding Substitutes (ICBS). Steps 1 and 2 are not included because they pertain to clinics’ policy and staff training, which are not directly observable to mothers and, thus, they would not be aware of or able to comment. The questionnaire consists of 24 multiple-choice questions with related sub-questions, for each of the “*10 Steps*”. For instance, two questions are used to assess the implementation of Step 4. The WHO/UNICEF self-assessment and monitoring questionnaire was translated into Greek by a team of specialists at the Attiko University Hospital under the supervision of Dr. Mexi Bourna (23). For the purposes of this study, the available Greek version was used, with the permission of the Greek National Commission of UNICEF.

### Definition of the 10 Steps variables

To determine the proportion of mothers who experienced each of the “10 Steps”, both individual component of a step (where applicable) and full implementation of the step were assessed. This approach was used to provide a more detailed depiction of which practices are sub-optimally implemented and facilitate comparisons with other studies that may have examined specific components separately. Some steps were operationalized with a single question; for instance, Step 9 -*no use of pacifier*, was considered to be implemented if mothers answered negatively to the question “*Has your baby sucked on a pacifier (dummy or soother), as far as you know, while you’ve been in the maternity unit?*”. Others required a combination of items to be fully assessed. For most questions, a positive answer indicates that the mother experienced that particular maternity practice. Supplementary Table S1 presents a detailed description of the operationalization of each of the 10 Steps.

### Estimation of the sum score of the 10 Steps

To investigate the relationship of the overall experience of the 10 Steps with BF and EBF, a sum score was also calculated, by assigning scores based on the implementation of each step. For binary variables, a score of zero was given for no implementation, and a score of two was given for full implementation. For categorical variables, no implementation was scored as zero, while partial implementation as one and full implementation as two. The sum scores were then categorized into quartiles to reflect the overall experience (theoretical range: 0–14). The lowest quartile (range: 0–4) comprised of mothers with the lowest sum scores, while the upper quartile included mothers with the highest sum scores (range: 7–14). The second quartile ranged between 4 and 5 and the third quartile between 5 and 7.

### Ethical considerations

Before commencing the study, all necessary approvals were obtained including approval from the Cyprus National Bioethics Committee and the Research Promotion Committee of the Ministry of Health. Furthermore, notification was sent to the Commissioner of Personal Data Protection. Permission to utilize all study tools was granted by the developers. Written informed consent was acquired from all mothers for their participation for each phase of the study. Confidentiality and anonymity were guaranteed. Mothers were informed that their participation was on a volunteer basis, that they could opt to participate in the baseline only if they did not wish to be contacted after their discharge and that they could withdraw their participation at any time point of the study.

### Statistical analysis

Summary statistics were used for the description of the socio-demographic characteristics of the participants at baseline. Differences in the prevalence of BF and EBF within the first 48 h after birth and at follow up were assessed using chi-square tests according to the self-reported experience of the “10 Steps” as measured at baseline, and the “10 Steps” Sum Scores,. Cramer V was used to assess the intercorrelation between the “10 Steps” variables. Logistic regression models were performed to examine the association of the self-reported experience of the “10 Steps” (independent variables) with BF and EBF initiation and continuation as dependent variables. Stepwise regression models were used in order to identify the association of the “10 Steps” with each of the outcomes of interest before and after mutually adjusting for each other.

## Results

### Participant characteristics

Out of a consecutive sample of 1,006 women initially approached, 797 met the eligibility criteria. The baseline sample included 586 women, corresponding to a response rate of 73.5%. Amongst the participants at the baseline phase, telephone contact was established with those who expressed the wish to also participate at the follow-up; specifically, 372 (response rate: 63.5%) were contacted by telephone at the first month, 383 at the fourth month and 340 at the sixth month, with very little drop-out between the three follow-up phases. More information on the process of participant selection is described in detail elsewhere (16). Supplementary Figure S1 shows a flow diagram of participation of mothers at each stage of the study.

Table 1 presents the socio-demographic profile of participants separately for the public and private sector. About 1 in 3 mothers who gave birth in the public sector were aged 18–24 years, in comparison to 20.9% in this age group among those who selected the private sector (*p* = 0.022, Chi square X^2^ = 13.197, d.f.= 5). The majority of mothers that gave birth at the private sector were in full-time employment, significantly higher than those who gave birth in the public sector (68.2% vs. 47.2%; *p* < 0.001, Chi square X^2^ = 50.312, d.f.= 3). The proportion of unemployed mothers that gave birth at the public sector was about three times higher than those that chose the private sector. There was evidence of a social gradient in favor of mothers of higher socioeconomic position, with mothers with higher educational attainment or higher family income being more likely to give birth at the private sector. With regards of country of origin, 76.2% of Cypriot mothers gave birth in the private sector in comparison with 63.3% in the public sector. With a high caesarean rate in the country, about half of the mothers who gave birth in the public sector had a vaginal birth, in comparison with only 36.9% of mothers in the private sector. Primiparous women were more likely to choose the private sector than multiparous (51.6% vs. 44.7%). Mean sum score for the overall “10 Steps” experience was low at 6.2 (SD = 2.7) with mothers giving birth in the public sector having a higher mean score than those who gave birth in the private sector (7.1; SD = 2.7 vs. 5.7; SD = 2.6; *p* < 0.001, *T*-test for equality of means = 6.044, d.f. = 584). Mothers who breastfed exclusively had generally higher scores, albeit still low, with a mean score of 8.5 (SD = 2.0) among women who EBF in the public sector in comparison to those who breastfed exclusively in the private sector (7.1; SD = 2.8).

**Table 1:** Socio-demographic characteristics and mean sum score of the “10 Steps” at (a) baseline and (b) between the public and private sector.

	Baseline		Public	Private	
	*N* = 586	%	% (*N*)	% (*N*)	p*
Age (years)^a^					
18–24	**145**	**24**.**7**	31.6 (67)	20.9 (78)	0.022
25–29	**258**	**44**.**0**	41.5 (88)	45.5 (170)	
30–34	**117**	**20**.**0**	18.4 (39)	20.9 (78)	
35–39	**28**	**4**.**8**	3.3 (7)	5.6 (21)	
≥40	**9**	**1**.**5**	2.4 (5)	1.1 (4)	
Not stated	**29**	**4**.**9**	2.8 (6)	6.1 (23)	
Education^a^					
Primary school	**4**	**0**.**7**	0.5 (1)	0.8 (3)	<0.001
Secondary school	**234**	**39**.**9**	53.8 (114)	32.1 (120)	
College/undergraduate	**183**	**31**.**2**	25.5 (54)	34.5 (129)	
Postgraduate degree	**124**	**21**.**2**	14.2 (30)	25.1 (94)	
Not stated	**41**	**7**.**0**	6.1 (13)	7.5 (28)	
Marital status^a^					
Married/cohabiting	**543**	**92**.**7**	91.1 (193)	93.6 (350)	0.006
Single	**12**	**2**.**0**	4.2 (9)	0.8 (3)	
Divorced/separated	**2**	**0**.**3**	0.9 (2)	0.0 (0)	
Not stated	**29**	**4**.**9**	3.8 (8)	5.6 (21)	
Employment status^a^					
Full Time	**355**	**60**.**6**	47.2 (100)	68.2 (255)	<0.001
Part Time	**65**	**11**.**1**	12.3 (26)	10.4 (39)	
Unemployed	**134**	**22**.**9**	38.2 (81)	14.2 (53)	
Not stated	**32**	**5**.**5**	2.4 (5)	7.2 (27)	
Monthly family net income^a^					
≤€1,500	**230**	**39**.**2**	57.5 (122)	28.9 (108)	
€1,501-€3,000	**192**	**32**.**8**	17.9 (38)	41.2 (154)	<0.001
€3,001-€4,500	**54**	**9**.**2**	3.8 (8)	12.3 (46)	
>€4,501	**17**	**2**.**9**	1.9 (4)	3.5 (13)	
Not stated	**93**	**15**.**9**	18.9 (40)	14.2 (53)	
Country of origin^a^					
Cypriot	**419**	**71**.**5**	63.2 (134)	76.2 (85)	<0.001
Not cypriot	**129**	**22**.**0**	32.5 (69)	16.0 (60)	
Not stated	**38**	**6**.**5**	4.2 (9)	7.8 (29)	
Type of birth^a^					
Vaginal	**249**	**42**.**5**	52.4 (111)	36.9 (138)	<0.001
Caesarian section	**320**	**54**.**6**	46.7 (99)	59.1 (221)	
Not stated	**17**	**2**.**9**	0.9 (9)	4.0 (15)	
Parity^a^					
Primiparous	**278**	**47**.**4**	40.1 (85)	51.6 (193)	0.001
Multiparous	**292**	**49**.**8**	59.0 (125)	44.7 (167)	
Not stated	**16**	**2**.**7**	0.9 (2)	3.7 (14)	
	**Mean (SD)**		**Mean (SD)**	**Mean (SD)**	*P* ^b^
Mean Score of the “10 Steps”	6.2 (2.7)		7.1 (2.7)	5.7 (2.6)	<0.001
Mean Score of the “10 Steps” for EBF within 48 h after birth	7.7 (2.5)		8.5 (2.0)	7.1 (2.8)	0.003

Bold values indicate statistical significance.

^a^ *p* of Chi-square test reported for categorical variables.

^b^ *p* of independent sample *t*-test reported for continuous variables.

### BF and implementation of the “10 Steps”

Table 2 presents the prevalence of breastfeeding initiation and continuation up to the 6th month after birth according to the self-reported experience of the “10 Steps for Successful Breastfeeding”.

**Table 2:** Prevalence of any breastfeeding initiation and continuation up to the 6th month according to self-reported experience of the “10 steps”*.*

WHO Steps for Successful Breastfeeding	*N*	48 h % BF (*N*)	*p*	*N*	1st month % BF (*N*)	*p*	*N*	4th month % BF (*N*)	*p*	*N*	6th month % BF (*N*)	*p*
		*N* = 494			*N* = 274			*N* = 169			*N* = 122	
Step 3: Information on BF prenatally												
Not implemented	339	82.9 (281)	0.143	219	78.1 (171)	0.080	228	46.9 (107)	0.134	215	35.3 (76)	0.283
Partially implemented	164	83.5 (137)		103	67.0 (69)		110	35.5 (39)		109	26.6 (29)	
Fully implemented	83	91.7 (76)		49	69.4 (34)		55	41.8 (23)		52	32.7 (17)	
Step 4: Early initiation of BF												
(a) Held the baby within 5 min												
Not implemented	266	75.6 (201)	**<0**.**001**	170	68.2 (116)	**0**.**025**	188	34.5 (61)	**0**.**001**	192	27.0 (47)	**0**.**024**
Implemented	320	91.6 (293)		201	78.6 (158)		227	50.5 (109)		225	37.1 (75)	
(b) Skin to Skin												
Not implemented	433	81.5 (353)	**0**.**002**	273	72.5 (198)	0.332	288	37.2 (107)	**<0**.**001**	276	29.3 (81)	**0**.**033**
implemented	153	92.2 (141)		92	77.5 (76)		105	59.0 (62)		100	41.0 (41)	
*(c) Both*												
Not implemented	464	81.5 (378)	**<0**.**001**	294	72.4 (213)	0.247	310	37.7 (117)	**<0**.**001**	299	29.4 (88)	**0**.**020**
Implemented	122	95.1 (116)		77	79.2 (61)		83	63.9 (53)		77	44.2 (34)	
Step 5: Help provided on BF												
(a) Help provided on BF												
Not implemented	90	44.4 (40)	**<0**.**001**	60	65.0 (39)	**0**.**088**	63	44.4 (28)	0.801	57	29.8 (17)	0.646
Implemented	496	91.5 (454)		311	75.6 (235)		330	42.7 (141)		319	32.9 (105)	
(b) Help provided on Baby positioning and attaching for BF												
Not implemented	86	44.2 (38)	**<0**.**001**	53	62.3 (33)	**0**.**038**	56	32.1 (18)	**0**.**076**	52	25.0 (13)	0.217
Implemented	500	91.2 (456)		318	75.8 (241)		337	44.8 (151)		324	33.6 (109)	
(c) Both												
Not implemented	122	54.1 (66)	**<0**.**001**	78	65.4 (51)	**0**.**055**	81	39.5 (32)	0.476	76	26.3 (20)	0.201
Implemented	464	92.2 (428)		293	76.1 (223)		312	43.9 (137)		300	34.0 (102)	
Step 7: Rooming in												
Not implemented	389	80.7 (314)	**0**.**001**	236	74.2 (175)	0.863	251	41.0 (103)	0.295	243	30.0 (73)	0.178
Implemented	197	91.4 (180)		135	73.3 (99)		142	46.5 (66)		133	36.8 (49)	
Step 8: Feeding on demand												
(a) Feeding every time the baby is hungry												
Not implemented	338	76.9 (260)		223	70.0 (156)		229	36.2 (83)		221	27.1 (60)	
Implemented	248	94.4 (234)	**<0**.**001**	148	79.7 (118)	**0**.**036**	164	52.4 (86)	**0**.**001**	155	40.0 (62)	**0**.**009**
(b) Feeding as long as the baby wants to												
Not implemented	273	75.8 (207)		168	72.6 (122)		181	42.5 (77)		170	30.0 (51)	
Implemented	313	91.7 (287)	**<0**.**001**	203	74.9 (152)	0.622	212	43.4 (92)	0.865	206	34.5 (71)	0.357
(c) Both												
Not implemented	413	80.4 (332)		263	70.7 (186)		273	38.5 (105)		294	83.7 (246)	0.676
Implemented	173	93.6 (162)	**<0**.**001**	108	81.5 (88)	**0**.**032**	120	53.3 (64)	**0**.**006**	114	43.0 (49)	0.004
Step 9: No use of pacifier												
Not implemented	294	83.7 (246)	0.676	188	127 (67.6)	**0**.**005**	195	36.9 (72)	**0**.**012**	188	25.0 (47)	**0**.**002**
Implemented	292	84.9 (248)		183	147 (80.3)		198	49.5 (98)		188	39.9 (75)	
Step 10: Guidance for seeking support												
Not implemented	442	81.4 (360)	**0**.**001**	279	72.8 (203)	0.403	289	40.1 (116)	**0**.**056**	273	30.0 (82)	0.104
Implemented	144	93.1 (134)		92	77.2 (71)		104	51.0 (53)		103	38.8 (40)	
ICBS												
Not implemented	145	69.7 (101)	**<0**.**001**	91	68.1 (62)	0.153	102	42.2 (43)	0.841	94	30.9 (29)	0.703
Implemented	441	89.1 (393)		280	75.7 (212)		291	43.3 (126)		282	33.0 (93)	

*P* of chi square analysis for categorical variables.

Bold values indicate statistical significance.

No association was found between BF initiation and the implementation of Step 3 (information on BF in the prenatal period). While women who reported receiving information about the benefits and management of BF in the prenatal period appeared slightly more likely to initiate BF while at the clinic, the difference was not statistically significant. Furthermore, the prevalence of breastfeeding in the longer term did not seem to differ according to whether women received information prenatally.

Implementation of Step 4, either partially or fully, appeared to be the most influential on breastfeeding initiation and continuation up to the sixth month. Only 20.8% of the participants have reported experiencing both of the components of Step 4 (i.e., *skin-to-skin*: 26.1%; *holding the baby within 5 min*: 54.4%). The majority of mothers who experienced either or both of the components of Step 4 indicated that they breastfed during their hospital stay. A statistically significantly lower prevalence of BF was recorded among those who reported first holding their newborn after *5 min*, at 75.6% (p: < 0.001; Chi square X^2^ = 28.092 d.f. = 1), or did not practice skin-to-skin, at 81.5% (*p* = 0.002’; 9.657 d.f.==1), or did not experience full implementation of step 4 (early initiation with skin-to-skin), at 81.5% (*p* < 0.001; Chi square X^2^ = 16.017 d.f. = 1). These differences according to whether Step 4 was practiced were evident with BF continuation up to the sixth month. About half of the mothers who *held their newborn within the first 5 min after birth* reported breastfeeding at the fourth month compared to 34.5% of those who did not (*p* = 0.001, Chi square X^2^ = 10.146 d.f. = 1).

A striking difference in the prevalence of BF was also recorded with 59.0% at the fourth month with mothers who experienced *skin-to-skin* compared to those who did not (59.0% vs. 37.2%; *p* < 0.001; Chi square X^2^ 16.364, d.f. = 1). The difference in BF prevalence was even more pronounced between mothers who experienced full implementation of Step 4 compared to those who reported that they did not. Specifically, 63.9% of mothers who reported full implementation of Step 4 at baseline were breastfeeding at the fourth month, which is nearly twice higher than the percentage (37.7%) of those who did not experience any of the components of Step 4 (*p* < 0.001, Chi square X^2^ = 20.234 d.f. = 1). At the sixth month, the prevalence of BF fell to 44.2% among mothers who experienced full implementation of Step 4. However, this rate was significantly higher than the 29.8% observed among those who reported no implementation of Step 4 (*p* = 0.020, Chi square X^2^ = 8.468, d.f. = 1).

Among mothers who received *help on BF* or practical assistance on *baby positioning and attaching (step 5)*, 91.5% and 91.2% initiated BF. This was about two times higher than those who did not experienced either of the two components of Step 5 with 44.4% and 44.2%, respectively (*p* < 0.001, Chi square X^2^ = 122.548, d.f. = 1). Differences in the prevalence of BF initiation appeared slightly smaller when both components were considered together. More than 90% of those experiencing full implementation of step 5, in comparison to 54.1% among those who did report any of the two components of Step 5 (*p* < 0.001, Chi square X^2^ = 122.548, d.f. = 1). A statistically significant difference between the individual components and the full implementation of Step 5 with BF continuation was also observed at the first month, but not at the 4th and 6th months.

At the first month, the prevalence of any BF among mothers who experienced either partial or full implementation of step 5 still appeared higher (Full implementation: 76.1% vs. 65.4%, *p* = 0.055, X^2^ = 3.669 df = 1; *help on positioning and attaching on BF*: 75.8% vs. 62.3%, *p* = 0.038, X^2^ = 4.302 df = 1; *help on BF*: 75.6% vs. 65.0%, *p* = 0.088, Chi square X^2^ = 2.906 d.f. = 1) At the fourth month, differences in the prevalence of BF persisted only for those who reported receiving practical assistance on BF positioning and attaching (44.8% vs. 32.1%; *p* = 0.078, Chi square X^2^ = 2.906, d.f. = 1). Differences were no longer evident at the sixth month.

The majority of mothers (91.4%) who experienced rooming-in initiated BF during hospital stay. This proportion was higher by comparison to those who did not report rooming-in (80.7%). The difference was statistically significant (p0.001, Chi square X^2^ = 11.209, d.f. = 1).

Implementation of Step 8 (*advice to breastfeed on demand*), either partially or fully, was found to be significantly associated with BF initiation with 94.4% vs. 76.9% comparing women who do and do not report receiving correct information about feeding frequency (*p* < 0.001; Chi square X^2^ = 32.842 d.f. = 1), 91.7% vs. 75.8% comparing women who do and do not report receiving correct information about feeding duration (*p* < 0.001, Chi square X^2^ = 27.6 d.f. = 1) and 93.6% vs. 80.4% (*p* < 0.001, Chi square X^2^ = 16.184 d.f. = 1)) comparing women who report receiving correct information about both aspects of feeding “on demand” compared to none. Differences in the prevalence of BF within the first six months persisted for women who reported full implementation of step 8, or at least among those reporting receiving the correct advice in terms of feeding frequency (*whenever the baby want to*), but not in terms of feeding duration (*for as long as the baby needs*). Among mothers who reported experiencing full implementation of Step 8 (advice for BF on demand—whenever and for as long as the baby wants), 42.2% also experienced rooming-in, while among those who experienced none of the two components of Step 8, the majority (70.0%) did not experience rooming-in (p0.004; data not shown; Chi square X^2^ = 8.095 d.f. = 1). These findings suggest of a positive intercorrelation between these two Steps (Cramer's V = 0.118, *p* = 0.04).

In contrast, while implementation of *Step 9* (*no use of pacifier*) did not appear to relate to initiation of breastfeeding while in the clinic (*p* = 0.676, Chi square X^2^ = 0.175 d.f. = 1), it was associated with the likelihood of longer breastfeeding duration (80.3% vs. 67.7% at the first month, *p* = 0.005 X^2^ = 7.837 df = 1; 49.0% vs. 36.4% at the fourth month, *p* = 0.012, Chi square X^2^ = 6.326 d.f. = 1; and 39.9% vs. 25.0% at the sixth month, p0.002, Chi square X^2^ = 9.513 d.f. = 1).

An association was also observed between the implementation of the ICMBS as well as Step 10 (advice about seeking help after discharge) with in-hospital BF initiation. Neither of these two aspects, however, seem to be associated with the prevalence of breastfeeding in the longer term.

### EBF and implementation of the steps

The prevalence of EBF initiation and continuation to the sixth month by the self-reported experience of the “10 Steps” while at the clinic are presented in Table 3. In general, the observed patterns of association between each of the “10 Steps” and the likelihood of exclusive breastfeeding are similar to those described above in the case of any breastfeeding. However, in the case of exclusive breastfeeding, even fewer aspects of the “good maternity” practices were found to be associated with EBF initiation and continuation up to the sixth month.

**Table 3:** Prevalence of exclusive breastfeeding initiation and continuation up to the 6th month according to self-reported experience of the “10 steps”*.*

WHO Steps for Successful Breastfeeding	*N*	48hours % (*N*)	*p* ^§^	*N*	1st month % (*N*)	*p* ^§^	*N*	4th month % (*N*)	*p* ^§^	*N*	6th month % (*N*)	*p* ^§^
		*N* = 110			*N* = 64			*N* = 51			*N* = 21	
Step 3: Information on BF prenatally												
Not implemented	339	18.3 (63)	0.252	219	19.6 (43)	0.343	244	13.4 (34)	0.465	256	5.7 (14)	0.735
Partially implemented	164	22.0 (36)		103	13.6 (14)		115	9.6 (11)		116	4.3 (5)	
Fully implemented	83	13.3 (12)		49	14.3 (7)		56	10.7 (6)		56	3.6 (2)	
Step 4: Early initiation of BF												
(a) Held the baby within 5 min												
Not implemented	266	12.4 (33)	**<0**.**001**	170	12.9 (22)	0.053	188	8.5 (16)	**0**.**036**	192	4.2 (8)	0.507
Implemented	320	24.1 |(77)		201	20.9 (42)		227	15.4 (35)		225	5.8 (13)	
(b) Skin to Skin												
Not implemented	433	13.9 (60)	**<0**.**001**	273	13.2 (36)	**0**.**001**	304	8.6 (26)	**<0**.**001**	307	3.3 (10)	**0**.**006**
Implemented	153	32.7 (50)		98	28.6 (28)		111	22.5 (25)		110	10.0 (11)	
(c) Both												
Not implemented	464	13.8 (64)	**<0**.**001**	294	12.9 (38)	**<0**.**001**	328	8.5 (28)	**<0**.**001**	332	3.6 (12)	**0**.**005**
Implemented	122	37.7 (46)		77	33.8 (26)		8	26.4 (23)		85	10.6 (10)	
Step 5: Help provided on BF												
(a) Help provided on BF												
Not implemented	90	21.1 (19)	0.537	60	18.3 (11)	0.808	67	17.9 (12)	0.126	67	4.5 (3)	0.820
Implemented	496	18.3 (91)		311	17.0 (53)		348	11.2 (39)		350	5.1 (18)	
(b) Help provided on Baby positioning and attaching for BF												
Not implemented	86	16.3 (14)	0.522	53	20.8 (11)	0.466	60	16.7 (10)	0.264	60	6.7 (4)	0.532
Implemented	500	19.2 (96)		318	16.7 (53)		355	41 (11.5)		357	7.8 (17)	
(c) Both												
Not implemented	122	20.5 (25)	0.584	78	17.9 (14)	0.854	87	14.9 (13)	0.396	87	4.6 (4)	0.834
Implemented	464	18.3 (85)		293	17.1 (50)		328	11.6 (38)		330	5.2 (17)	
Step 7: Rooming in												
Not implemented	389	43 (11.1)	**<0**.**001**	236	16.5 (39)	0.625	267	11.6 (31)	0.572	270	4.1 (11)	0.223
Implemented	197	67 (34.0)		135	18.5 (25)		148	13.5 (20)		147	6.8 (10)	
Step 8: Feeding on demand												
(a) Feeding every time the baby is hungry												
Not implemented	338	15.4 (52)	**0**.**014**	223	13.5 (30)	**0**.**017**	247	10.1 (25)	0.103	249	5.2 (13)	0.834
Implemented	248	23.4 (58)		148	23.0 (34)		168	15.5 (26)		168	4.8 (8)	
(b) Feeding as long as the baby wants to												
Not implemented	273	16.1 (44)	0.124	168	16.1 (27)	0.584	189	21 (11.1)	0.504	189	3.7 (7)	0.257
Implemented	313	21.1 (66)		203	18.2 (37)		226	13.3 (30)		228	6.1 (14)	
(c) Both												
Not implemented	413	15.5 (64)	**0**.**002**	263	14.4 (38)	**0**.**026**	293	10.9 (32)	0.188	294	4.8 (14)	0.692
Implemented	173	26.6 (46)		108	24.1 (26)		122	15.6 (19)		123	5.7 (7)	
Step 9: No use of pacifiers												
Not implemented	294	10.2 (30)	**<0**.**001**	188	8.0 (15)	**<0**.**001**	209	8.6 (18)	**0**.**022**	210	2.4 (5)	**0**.**013**
Implemented	292	27.4 (80)		183	26.8 (49)		206	33 (16.0)		207	7.7 (16)	
Step 10: Guidance for seeking support												
Not implemented	442	18.1 (80)	0.466	279	18.6 (52)	0.218	304	12.2 (37)	0.903	304	5.6 (17)	0.394
Implemented	144	20.8 (30)		92	13.0 (12)		111	12.6 (14)		113	3.5 (4)	
ICBS												
Not Implemented	145	14.5 (21)	0.127	91	17.6 (16)	0.923	109	13.8 (15)	0.586	111	7.2 (8)	0.222
Implemented	441	20.2 (89)		280	17.1 (48)		306	11.8 (36)		306	4.2 (13)	

Bold values indicate statistical significance.

^§^ *p* of Chi square analysis; The term “good advice” refers to guidance that successfully facilitates the implementation of the “10 Steps”, while “bad advice” refers to any guidance that fails to lead to the implementation of these Steps.

No association was found between Step 3 (information on breastfeeding in the prenatal period) and exclusive breastfeeding initiation and continuation. Similarly, no associations with EBF were observed with Step 5 (offered practical assistance), Step 10 (advice on seeking help in the community) and the implementation of the ICMBS.

The prevalence of EBF initiation was significantly higher among mothers who experienced partial or complete implementation of *Step 4* (early contact with baby with skin-to-skin). The proportion of mothers who initiated EBF within the first 48 h was about 2-times higher among those who held their infant *within the first 5 min after birth* (24.1%) in comparison to those that did not (12.4%; *p* < 0.001, Chi square X^2^ = 12.944 d.f. = 1). Among those who experienced skin-to-skin, 32.7% breastfeed their baby exclusively during their stay at the clinic, which was about twice higher than those who did not (13.9%; *p* < 0.001, Chi square X^2^ = 26.269 d.f. = 1). The difference was more striking for full implementation of Step 4 since as many as 37.7% vs. 13.8% of women reported breastfeeding their baby exclusively while at the clinic (*p* < 0.001, Chi square X^2^ = 37.625 d.f. = 1).

This difference in the likelihood to be breastfeeding exclusively persisted up to the sixth month among mothers who had *skin-to-skin* and full implementation of *Step 4*. Mothers who experienced skin-to-skin were consistently about two to three times as likely to be breastfeeding exclusively in the longer term compared to those who did not (1st month: 28.6% vs. 13.2% *p* < 0.001, Chi square X^2^ = 11.957 d.f. = 1; 4th month:22.5% vs. 8.6%, *p* < 0.001, X^2^ = 14.722 df = 1; 6th month: 10.0% vs. 3.3% *p* = 0.006, Chi square X^2^ = 7.699 d.f. = 1). The differences were even stronger when full implementation of step 4 was considered (1st month: 33.8% vs. 12.9%, *p* < 0.001, Chi square X^2^ = 19.675 d.f. = 1; 4th month:26.4% vs. 8.5%, *p* < 0.001, Chi square X^2^ = 22.408 d.f. = 1; 6th month: 10.6% vs. 3.6% *p* = 0.005, Chi square X^2^ = 7.964 d.f. = 1).

EBF initiation was also associated with receiving correct advice in terms of feeding frequency (“*any time the baby wants to”*) and full implementation of Step 8 (*feeding on demand*), but not with good advice with regards to feeding duration alone (“*as long as the baby wants to”*). Any effect on “good advice” with regards to full implementation of Step 8 was evident only at the first month. Beyond that, the prevalence of women exclusively feeding their infants at the fourth and sixth month did not differ according to whether the reported being given advice on feeding on demand while at the clinic.

In relation to rooming-in, women who practiced it were three times more likely to be exclusively breastfeeding while at the clinic (34.0% vs. 11.1%; *p* < 0.001, Chi square X^2^ = 45.197 d.f. = 1). However, any association between rooming-in and exclusive breastfeeding was restricted to the first 48 h, as was the case with any breastfeeding. The likelihood of women exclusively breastfeeding in the longer term did not differ according to whether they roomed-in with their infants while at the clinic.

While at the clinic, women who did not report using pacifiers, were nearly three times more likely to be exclusively breastfeeding (27.4% Vs 10.2; *p* < 0.001, Chi square X^2^ = 28.401 d.f. = 1). Similarly, women who did not report using pacifiers while at the clinic, were at least twice as likely to be exclusively breastfeeding at the first, fourth and sixth month.

### Prevalence of EBF by the sum score of the “10 Steps”

As shown in Table 4, both EBF initiation as well as continuation up to the sixth month showed a clear stepwise increasing pattern in terms of the overall experience of the “10 Steps” as depicted by the pattern of association observed across quartiles of mothers with increasing “10 Steps” sum score. The quartile of mothers with the highest sum scores were more likely to be breastfeeding exclusively during their stay in the maternity clinic (34.2%). In contrast, among the quartile of mothers with the lowest BSES scores only 7.5% initiated EBF (*p* < 0.001, Chi square X^2^ = 11.904 d.f. = 6). The prevalence of EBF for the two middle groups (2nd and 3rd quartiles) appeared in-between with 14.4% and 19.0%, respectively.

**Table 4:** Prevalence of breastfeeding and exclusive breastfeeding at the first 48 h as well as at the first, fourth and sixth month according to quartiles of increasing levels of the sum score of implementing of the “10 Steps” for Successful Breastfeeding as experienced by mothers within 48 h after birt*h.*

			24 h				1st month				4th month				6th month	
		No BF	Mixed BF	EBF		No BF	Mixed BF	EBF		No BF	Mixed BF	EBF		No BF	Mixed BF	EBF
	*N*	% (*N*)	% (*N*)	% (*N*)	*N*		% (*N*)	% (*N*)	*N*	% (*N*)	% (*N*)	% (*N*)	*N*	% (*N*)	% (*N*)	% (*N*)
Lower Quartile (range:0.00–4.00)	147	40.8 (60)	51.7 (76)	7.5 (11)	90	33.3 (30)	54.4 (49)	12.2 (11)	93	62.4 (58)	23.7 (22)	14.0 (13)	89	71.9 (64)	24.7 (22)	3.4 (3)
2nd Quartile (range: 4.00–5.00)	146	10.3 (15)	75.3 (110)	14.4 (21)	97	27.8 (27)	58. (57)	13.4 (13)	95	63.2 (60)	27.4 (26)	9.5 (9)	90	71.9 (64)	24.7 (22)	3.4 (3)
3rd Quartile (range: 5.00- 7.00)	147	7.5 (11)	75.3 (108)	19.0 (28)	88	22.7 (20)	54.5 (48)	22.7 (20)	98	58.7 (57)	28.6 (28)	13.3 (13)	95	74.4 (67)	1.9 (17)	6.7 (6)
Upper Quartile (range: 7.00- 14.00)	146	4.1 (6)	61.6 (90)	34.2 (50)	94	20.8 (20.8)	58.3 (56)	20.8 (20)	106	44.3 (47)	40.6 (47)	15.1 (16)	101	53.5 (54)	40.6 (41)	5.9 (6)
*P*			**<0.001**				**0.240**				**0.093**				**0.014**	
*p* for trend			**<0.001**				**0.011**				**0.034**				**0.015**	

Sum Score min-max: 0 (implementation of no step) −14 (all Steps experienced); Scores of binary variables: 0 (no implementation) and 2 (full implementation); Scores of categorical variables: 0 (no implementation), 1 (partial implementation) and 2 (full implementation); Sample Size at each time point: *Ν* = 586 during the first 48 h, *Ν* = 372 at 1st month, *Ν* = 415 at 4th month and *Ν* = 417 at 6th month; *p* of Chi-square test of independence and chi-square test for trend (Linear-by-Linear) is reported.

Bold values indicate statistical significance.

At the first month, there were almost two times as many mothers exclusively breastfeeding among those at the upper quartile of the “10 Steps” sum score compared to the lower quartile (20.8% vs. 12.8%, *p* = 0.240, Chi square X^2^ = 7.891 d.f. = 6), with a stepwise association of the sum score and EBF across quartiles (linear-by-linear chi-square *p* = 0.011). Differences in the prevalence of EBF at the fourth month were narrower, as 15.1% of mothers at the upper quartile of the overall experience were exclusively breastfeeding compared to 14.0% of women at the lower quartile (*p* = 0.093, Chi square X^2^ = 10.859 d.f. = 6). By the sixth month, fewer than 10% of the mothers at the upper quartile were exclusively breastfeeding, in comparison to only 3.4% among those at the lowest quartile (*p* = 0.014, Chi square X^2^ = 15.918 df = 6).

### Association of the “10 Steps” to Successful Breastfeeding with breastfeeding and exclusive breastfeeding

#### EBF

Table 5 presents the results of the stepwise Logistic regression analysis of the likelihood to EBF at 48 h and up to the sixth month according to the implementation of the “10 Steps”, while mutually adjusting for each other.

**Table 5:** Stepwise logistic binary regression analysis of EBF at 48 h and up to the sixth month with regards to the implementation of the “10 Steps*”.*

	48 h		1st month		4th month		6th month	
	AdjOR (95% CI)	*p*	AdjOR (95% CI)	*p*	AdjOR (95% CI)	*p*	AdjOR (95% CI)	*p*
Step 3: Information on BF prenatally								
None	1.00							
Partial	1.15 (0.69, 1.90)	0.597						
Full	0.37 (0.17, 0.81)	**0**.**012**						
Step 4: Early initiation of BF								
None	1.00		1.00		1.00			
Partial	1.19 (0.68, 2.08)	0.537	0.945 (0.47, 1.90)	0.874	1.22 (0.55, 2.75)	0.624		
Full	3.75 (2.09, 6.72)	**<0**.**001**	2.87 (1.44, 5.71)	**0**.**003**	4.97 (2.27, 10.88)	**<0**.**001**		
Step 5: Help provided on BF								
None					1.00			
Partial					0.24 (0.06, 0.88)	**0**.**032**		
Full					0.30 (0.12, 0.72)	**0**.**008**		
Step 7: Rooming in								
No	1.00							
Yes	3.47 (2.18, 5.51)	**<0**.**001**						
Step 8:								
None								
Partial								
Full								
Step 9: No Use of a Pacifier								
No	1.00		1.00				1.00	
Yes	2.50 (1.54, 4.07)	**<0**.**001**	3.79 (2.02, 7.13)	**<0**.**001**			3.44 (1.23, 9.56)	**0.018**

All Steps were included in the model with the exception of Step 6 (exclusive breastfeeding); only Steps remaining in the final stepwise regression model are shown, with mutual adjustment for each other.

Bold values indicate statistical significance.

Full implementation of both components of Step 4 appeared to have a strong association with EBF initiation at baseline with adjOR = 3.75 (95% CI:2.09, 6.72; *p* = <0.001), but this was not apparent for partial implementation of Step 4 (adjOR = 1.19, 95% CI:0.68, 2.08; *p* = 0.537). The association with full, but not partial, implementation, of Step 4 was still evident at the first month (adjOR: 2.87; 95% CI: 1.44, 5.71, *p* = 0.003) and at the fourth month. Specifically, women who reported full implementation of step 4 were about 5-times more likely to be exclusively breastfeeding at the fourth month (adjOR: 4.97, 95% CI: 2.27, 10.88; *p* < 0.001).

On the other hand, rooming-in appears to be associated with EBF initiation (adjOR: 3.47, 95% CI: 2.18, 5.51; *p* < 0.001), but not with the likelihood that a mother will continue exclusively breastfeeding in the longer term. However, among mothers who reported experiencing full implementation of Step 8 (*advice for BF on demand—whenever and for as long as the baby wants*), 42.2% also experienced rooming-in, while among those who experienced none of the two components of Step 8, the majority (73.7%) did not experience rooming-in (*p* = 0.005; data not shown).

The implementation of Step 9 (no use of pacifiers) was positively associated with EBF initiation, as well as continuation of exclusive breastfeeding. Mothers who practiced Step 9 during their hospital stay were approximately twice as likely to initiate exclusive breastfeeding and 3–4 times more likely to continue exclusive breastfeeding at the first (adjOR: 3.79, 95%: 2.02, 7.13; *p* < 0.001) and sixth months (adjOR: 3.44; 95%: 1.23, 9.56; *p* = 0.018).

Interestingly, while an association was observed between Step 3 (information on the benefits and management of breastfeeding in the prenatal period) and EBF initiation [OR = 2.24, 95% CI 0.98, 5.11; *p* = 0.055 (data not shown)] before considering the other variables, this association reversed in the adjusted models (which retain Step4: early contact with skin-to-skin, step 7: rooming-in and Step 9: no use of pacifiers as most predictive of EBF initiation).

#### BF

Table 6 shows the stepwise regression models for the association between BF at 48 h as well as at the first, fourth and sixth months with self-reported experience of the “10 Steps”.

**Table 6:** Stepwise logistic binary regression analysis of BF at 48 h and up to the sixth month with regards to the implementation of the “10 Steps*”.*

	48 h		1st month		4th month		6th month	
	AdjOR (95% CI)	*p*	AdjOR (95% CI)	*p*	AdjOR (95% CI)	*p*	AdjOR (95% CI)	*p*
Step 3								
None			1.00					
Partial			0.54 (0.31, 0.92)	**0**.**023**				
Full			0.56 (0.28, 1.14)	0.107				
Step 4: Early initiation of BF								
None	1.00				1.00			
Partial	1.83 (0.99, 3.36)	**0**.**051**			1.52 (0.95, 2.40)	0.079		
Full	4.23 (1.62, 11.00)	**0**.**003**			3.60 (2.06, 6.30)	**<0**.**001**		
Step 5: Help provided on BF								
None	1.00		1.00					
Partial	8.61 (3.29, 22.52)	**<0**.**001**	2.01 (0.76, 5.33)	**0**.**161**				
Full	21.72 (9.69, 48.69)	**<0**.**001**	2.86 (1.32, 6.18)	**0**.**008**				
Step 8: Feeding on demand								
None	1.00						1.00	
Partial	2.88 (1.49, 5.55)	**0**.**002**					0.82 (0.47, 1.42)	0.479
Full	3.62 (1.69, 7.78)	**0**.**001**					1.77 (1.03, 3.03)	**0**.**038**
Step 9: No use of pacifier								
No			1.00				1.00	
Yes			2.09 (1.28, 3.41)	** 0**.**003**			2.01 (1.28, 3.14)	**0**.**002**
ICBS								
No	1.00							
Yes	2.85 (1.57, 5.16)	**0**.**001**						

All Steps were included in the model with the exception of Step 6 (exclusive breastfeeding); only steps remaining in the final stepwise regression model are shown, after mutual adjustment for each other.

Bold values indicate statistical significance.

Implementation of Step 5 (practical assistance with breastfeeding) appeared to have the greatest effect on BF initiation than any other step both before (not shown) and after considering all others in a stepwise model. Mothers who experienced either partial, and even more so full, implementation of this Step were 8–22 times more likely to initiate BF (adjOR: 8.61, 95% CI: 3.29, 22.52, *p* < 0.001 at partial and adjOR: 21.72, 95% CI: 9.69, 48.69, *p* < 0.001 at full implementation).

As with the case of exclusive breastfeeding, implementation of Step 4 (partial or full) was positively associated with BF initiation as well as continuation. Mothers who experienced one of the two components of Step 4 were about twice more likely to initiate BF (adjOR: 1.83 (95% CI: 0.99, 3.36, *p* = 0.051). Mothers who experienced any (but not both) of the two components were 1.52-times more likely to breastfeed (95% CI: 1.39, 4.74, *p* = 0.003). Full implementation of Step 4 (i.e., *holding baby within 5 min* and *practicing skin-to-*skin) was one of the most predictive variables of the likelihood a mother would breastfeed in the longer term. Mothers who reported skin-to-skin within the first 5 min were at least 3 times to breastfeed at the fourth month (adjOR: 3.60 95% 2.06. 6.30, *p* < 0.001), even though no association was observed at the first and sixth month.

Implementation of Step 8 (*advice on how to breastfeed on demand*), whether reported as partially or fully implemented, also appeared to be associated with BF initiation with adjOR: 2.88 (95% CI: 1.491, 5.55) and adjOR: 3.623 (95% CI: 1.69, 7.78); *p* = 0.001) respectively. While being advised to BF on demand did not seem to predict whether a woman would be breastfeeding at the first and fourth month, it appears that this was more predictive whether a woman would be breastfeeding at the sixth month, even more so than step 4 (which was more predictive of breastfeeding behaviour at the first and fourth month). Specifically, the only variables that were predictive of breastfeeding at the sixth month were Step 8 along with Step 9 (no pacifiers). Women who reported full implementation of Step 8 were more likely to be breastfeeding at the sixth month (adjOR: 1.77, 95% CI: 1.03, 3.03, *p* = 0.038).

In relation to Step 9 (no pacifiers), mothers who did not report using them were about two times more likely to breastfeed at first and sixth month. In fact, similarly, to breastfeeding on demand, the use of pacifiers was more predictive than other variables of whether a woman would be still breastfeeding at the sixth month. No significant differences in the ORs were observed before and after adjusting for the other Steps, which suggests that the practice of not using pacifiers while at the clinic is an independent predictor of BF continuation (data not shown).

Finally, a positive association was found between the implementation of ICBS and BF initiation (adjOR: 2.85 95% CI: 1.57, 5.16, *p* = 0.001). Nevertheless, this did not predict whether a woman would be breastfeeding in the longer term.

Overall, it appears that Steps 4 (early contact with skin-to-skin), 8 (advice by the staff to BF on demand), 9 (no use of pacifiers) are more predictive of whether a woman will initiate breastfeeding. Step 5 (practical assistance) is added to this list of strong predictors, even though, this may be an artefact since no assistance would be expected to be provided to women who express the wish not to breastfeed. Furthermore, the implementation of the Code appears to be an independent predictor of breastfeeding initiation; however this does not seem to be predictive of longer-term breastfeeding outcomes. Among these variables, only three Steps (4, 8 and 9) in some combination appeared to be more consistently predictive of the likelihood a mother would be breastfeeding at the first, fourth and sixth month.

## Discussion

### Main findings

The objective of the present study was to assess the effect of maternity practices on BF and EBF initiation, continuation and exclusivity up to the sixth month. Early initiation with skin-to-skin (Step 4) and avoidance of pacifiers (Step 9) appear to be the two practices that are more consistently and strongly associated with a mother's likelihood to initiate and continue to exclusively breastfeed up to the 4th or 6th month. In fact, the association of Step 4 with EBF initiation and continuation appears to be stronger when fully implemented, suggesting a synergistic effect of both components (i.e., early initiation of BF and skin-to-skin). Furthermore, no use of pacifiers while at the clinic appears to be related with BF continuation, even if not exclusively. In contrast, feeding on demand (Step 8) and rooming-in (Step 7) appear to be predictive of EBF initiation, but it does not seem to be equally predictive continuation of EBF in the long term. In addition, while assistance by staff (Step 5) is related to BF initiation and continuation, it does not appear to be predictive of exclusive breastfeeding.

### The implementation of the “10 Steps” and BF and EBF initiation

Implementation of Step 5 (assistance by maternity clinic staff) appears to have the greatest effect on BF initiation, whereas no association was found with EBF initiation. This seemingly paradoxical result might be due to the fact that mothers who initiated BF (but are not breastfeeding exclusively) might have actually experienced problems and requested for maternity staff support. Furthermore, the association is more likely to be bi-directional as to some extent mothers who intent to breastfeed are more likely to request assistance compared to mothers who had decided they would not breastfeed their infants. Thus, practical support and encouragement provided by the maternity clinic staff might have helped them to overcome potential problems during the first stages of BF initiation. In fact, this assistance might not necessarily pertain to breastfeeding support, but assistance with formula feeding. It is of note that 82.6% of the mothers who did not breastfeed exclusively within the first 48 h reported introducing formula supplementation. Of those, 84.2% reported receiving help from the maternity staff. It is likely that women who decided that they would not breastfeed at all, were simply not offered assistance by the maternity clinic staff. Based on our findings, practical assistance with BF by the maternity clinic staff was only predicting that a woman will initiate breastfeeding, and not whether she will exclusively breastfeed, as no association was observed between Step 5 and EBF.

Furthermore, Step 8 (breastfeeding on demand) and ICMBS were also associated with BF initiation, but not EBF. With regard to Step 8, it has been previously suggested that breastfeeding on demand might contribute to fewer formula feedings and thus might contribute to successful BF establishment (24). With regards to the ICMBS, it should be noted that a high proportion of mothers in this study reported not receiving any breast milk substitutes at least up to the point of the interview i.e., 24–48 h after birth. While this suggests a high level of implementation of the ICMBS, it might be an overestimation as a result of the timeframe. Commonly free samples of breast milk substitutes and other products may be given at discharge (usually the third day as per current practices in Cyprus).

In contrast to the above, rooming-in appears to be positively associated with exclusivity. Mothers who roomed-in with their infants are not necessarily more likely to have breastfed, but if they did, breastfeeding was exclusive. Even though in terms of its clinical significance, it corresponds to a difference of only one additional mother initiating breastfeeding for every ten mothers rooming-in, this suggests that while mothers who room-in are more likely to breastfeed, the prevalence of breastfeeding initiation was also relatively high even among mothers who did not room-in with their infants. Continuous mother-infant cohabitation may facilitate the implementation of other maternity practices, and in particular BF on demand. This is supported by our findings which suggest that there is an intercorrelation between rooming-in and full implementation of Step 8. The implementation of Step 8 (i.e., truly unrestricted feeding) is only feasible with rooming-in (25). On the other hand, it may also be suggestive of a possible bi-directional association whereby women who intent to breastfeed exclusively may actively and more assertively request to room-in with their infant in order to breastfeed on demand. While the study design does not allow any inferences about the direction of association, this association may to some extent reflect the mothers’ decision to maximize the chances of exclusive breastfeeding by ensuring that no other liquids are given to the infant during their time in the clinic, especially in the context of Cyprus, where rooming-in is not the default practice. In many clinics, infants are kept in a nursery and care is provided by the midwives in order to allow mothers to rest after birth, expect if it is requested by the mother.

Existing literature presents conflicting evidence on rooming-in and BF initiation. Some studies reported a positive association between rooming-in and BF (25), while others have not (9). However, there is evidence to suggest that rooming-in, through supporting the implementation of Step 8 (26), is linked to longer breastfeeding duration (27), more frequent feedings (28), earlier breast milk production and exclusivity (27). Infants who room-in with their mothers tend to cry less, soothe more easily and sleep longer (27). This may reduce the risk of pacifier to soothe the infant, thereby minimizing breastfeeding difficulties and maintaining milk production (27). However, out findings suggest that the effect of rooming-in did not persist on BF continuation, which aligns with findings from other studies (9, 29). This might be due to the fact that other BF determinants, such as BF difficulties encountered after discharge, the lack of social support, concerns about insufficient milk production, may have a stronger influence on BF discontinuation.

With regard to Step 4, our findings suggest a synergistic effect of both components. The available evidence in the literature mainly focuses on the investigation of the individual effects of each component i.e., ’*skin-to-skin’* (30–35) and “*initiation of BF within one hour after birth*” (36, 37), with studies confirming the beneficial effect of each on BF and EBF initiation. In particular, research on skin-to-skin, suggests a dose-response relationship, indicating that longer durations of skin-to-skin contact are more strongly associated with the likelihood of initiating exclusive breastfeeding (33, 34). Conversely, shorter durations of skin-to-skin contact at birth have been linked to an increased risk of early introduction of formula feeding (38). Mothers who engage skin-to-skin contact are more likely to initiate BF early (39), which in turn leads to successful suckling and increases the chances of initiating BF without assistance (40).

Delivery by C/S may influence the successful implementation of Step 4, not allowing the implementation of skin-to-skin. The high rate of C/S in our sample would explain to some extent the low proportion of mothers reporting practicing skin-to-skin. Nevertheless, even among mothers who gave birth vaginally, only 36.5% reported experiencing skin-to-skin, compared to 19.4% who delivered by C/S. A more striking difference by mode of delivery was observed in terms of early initiation, since 93.2% of mothers who gave birth vaginally reported holding the baby within the first one hour (of whom, 35.7% experienced full implementation of Step 4) in comparison with 58.1% of the mothers who delivered by cesarean section (of whom, 13.4% experienced full implementation of Step 4).

Finally, with regard to Step 3 it is not entirely clear what might explain the reversal of the association (adjOR = 0.37, 95% CI 0.17, 0.81) once the most predictive variables are included in the model. Since the variable refers to information being given in the prenatal period, it may suggest that among women who practiced skin-to-skin, rooming-in and use no pacifiers, it is the ones who did not attend prenatal classes (where such information is more likely to have been provided) who were more likely to initiate exclusively breastfeeding.

### The implementation of the “10 Steps” and BF and EBF duration

In contrast, and similarly to the case of any breastfeeding, the association of Step 9 (no use of pacifiers) with EBF does not seem to be restricted to the first 48 h, but appears to persist for the first six months. This would suggest that women who reported not using pacifiers while at the clinic were not only more likely to initiate exclusive breastfeeding, but they were also more likely to be EBF in the longer term. This association has been confirmed by other studies (9, 41–46).

It has been suggested that mothers who introduce pacifiers early after birth are more likely to perform infrequent (42) and shorter feedings (41) which, in turn, might result to insufficient production of breast milk (42). Other studies suggested that pacifier use may be an independent determinant of BF discontinuation, over and above the existence of BF problems or incorrect BF techniques (445). Alternatively, it has been suggested by O’ Connor *et al*. (2009) that the association between BF and pacifier use can be attributed to residual confounding and reverse causality (47). For example, intention to breastfeed exclusively might influence mothers to avoid pacifier use in general, including for infant calming and soothing. At the same time, use of a pacifier might be a marker for the presence of BF problems or the beginning of weaning (i.e., reverse causality). In fact, they describe pacifiers as “*an implicit form of weaning when mothers are ambivalent about breastfeeding*” (47). Pacifier use might also be regarded by mothers as a positive behavior associated with longer intervals between feedings and taking the babies of their breast increasing the risk of insufficient milk production (residual confounding) (47). Relevant randomized controlled trials revealed no difference in BF duration between different pacifier interventions (i.e., delayed introduction of pacifiers, educational programme for alternative methods of soothing) supporting that pacifier use might be an indicative maker of more complex behaviors (47).

In this study, no use of pacifiers while at the clinic (i.e., implementation of Step 9) was associated with continuation of EBF up to the first month. However, this association was not maintained at fourth and sixth month. A number of observational studies have demonstrated an association between pacifier use and premature discontinuation of BF (41, 42, 47) or non-exclusivity (41, 48). In fact, use of pacifiers was found to be independently associated with shorter BF duration (42). This observation might come into agreement with the potential explanation suggested that pacifier use does not have a direct effect on early BF discontinuation rather than it is an indicator of a number of factors and behaviors that are might influence BF outcomes (49, 50). For example, mothers who experience breastfeeding difficulties and decide to discontinue BF or EBF, might initiate pacifier use to facilitate the introduction of bottle feeding (12). In an RCT examining the impact of breastfeeding technique on breastfeeding duration, it was found that only ineffective sucking (adjHR: 1.87; 95% CI: 1.38–2.55) and ineffective milk transfer (adjHR: 1.78; 95% CI: 1.29–2.45) significantly affected the duration of breastfeeding. Positioning and latching did not appear to influence breastfeeding duration, and notably, pacifier use was not found to have an effect on breastfeeding technique (48). A recent systematic- review of RCTs confirms that pacifier use may not have an association with breastfeeding duration and exclusivity, which comes into contradiction with findings of the previous observational studies, suggesting of no causal association with breastfeeding (51). Mothers who successfully overcome breastfeeding challenges and choose to continue exclusive breastfeeding, the use of a pacifier is less likely to affect the duration of breastfeeding. Other studies, however, showed of no association (52, 53).

In this study, only full implementation of Step 4 was associated with BF and EBF continuation. This observation suggests the importance of successful BF initiation by the implementation of early skin-to-skin to the successful BF and EBF establishment and continuation (35, 46). Early skin-to-skin was associated with the experience of fewer BF difficulties such as breast engorgement (35), and thus with reduced risk of formula introduction or early weaning (26).

In general, it should be noted that the association between BF initiation and exclusivity and the experience of any of the Steps is likely to be bi-directional. Mothers who are more knowledgeable on breastfeeding issues and those who intent to breastfeed, and even more so exclusively, might be more likely to request the implementation of the Steps (51), indicating a bi-directional association between BF and the “10 Steps”. In this case, maternity practices might be used as facilitators in successful BF initiation, continuation and exclusivity with the reduction of the risk of BF difficulties and, the development of maternal skills in infant care and enhancement of BFSE.

### Strengths and limitations

A clear strength of the study is its prospective design which facilitated the assessment of infant feeding practices over the period of the first six months, avoiding the recall bias of a retrospective design. It should be acknowledged that the present study measured the individual association of each of the “10 Steps” on BF outcomes and on breastfeeding self-efficacy, but not the cumulative effect of all maternity practices as a pack on BF outcomes. Nevertheless, the findings suggest that a better overall experience of the “10 Steps”, as operationalized by a sum score, was associated with a higher likelihood of BF and EBF initiation and continuation, even though the overall scores were relatively low. IT is of note that only 1% of the sample reported experiencing all the Steps, even when not including Step 6—Exclusive Breastfeeding. The study applied stepwise regression models in order to identify the set of practices which are more predictive of BF outcomes and breastfeeding self-efficacy. This approach was preferred to a multivariable model (which would include all variables) due to collinearity between the variables.

Recognizing the potential bi-directional association between experiencing the “10 Steps”, breastfeeding self-efficacy and BF behavior/outcomes, the study avoided hypothesizing a causal relationship between the experience of “good practices” and positive BF outcomes, which may also be mediated by breastfeeding self-efficacy. The study described the magnitude of the observed associations. It is, thus, recognized that while the experience of “good practices” may positively impact maternal breastfeeding self-efficacy, at the same time, mothers with higher breastfeeding self-efficacy may be more likely to request and demand the implementation of certain “good practices”. Similarly, while the experience of “good practices” may positively impact on BF outcomes, both short-term as well as long-term, at the same time, intention to breastfeed (measured here only with a single-item) or other potential covariates related to motivation for BF (e.g., beliefs and attitudes related to breastfeeding and child-rearing, maternal personality characteristics etc.) may actually be driving both the likelihood that a mother will breastfeed and/or breastfeed exclusively as well as the likelihood that she will implement or demand the implementation of certain “good practices”.

Furthermore, the study focused on collecting data regarding the implementation of certain practices within the first 24–48 h. While this may be a critical period that may influence long-term practices (for instance, with regard to rooming-in at home and no use of pacifiers). no data were collected on these practices beyond the maternity clinic stay at the follow-up.Furthermore, the specific study focuses on only one aspect of the multidimensional issue of breastfeeding, as outlined by the socio-ecological model. It does not explore other key factors that influence breastfeeding initiation and continuation, such as social support and cultural norms.Finally, the study used only quantitative methods since the aim was to provide first-time estimates of breastfeeding in Cyprus beyond the 48 h and explore the extent to which the implementation of the “10 Steps” are associated with breastfeeding outcomes. However, an in-depth exploration of the perceptions and attitudes of women in Cyprus with regards to breastfeeding, and perceived reasons for premature discontinuation using qualitative methods would have enriched the study findings.

## Conclusions

The findings of the present study indicate the importance of the “10 Steps” to support successful BF initiation, continuation and exclusivity. While the study findings cannot delineate a possible bi-directional and cumulative effect between the “10 Steps” and breastfeeding, full implementation of Step 4 (early initiation with skin-to-skin) appears to have the strongest association with breastfeeding initiation, continuation and exclusivity. Other Steps may influence either breastfeeding initiation or continuation, suggesting their complementarity and indicating the importance of the total adherence to these maternity practices.

This study showed that the overall adherence to the “10 Steps” was low, with only 0.3% of mothers reporting that they experienced all the Steps. This suggests a lack of established protocols in maternity clinics and calls for the need to establish monitoring mechanisms and continuous education and professional development training of healthcare staff in the implementation of the “10 Steps”. Even though, the National Breastfeeding Committee of the Cyprus Ministry of Health has developed a national strategy for breastfeeding (2011) and Policy (2015) which includes a call to launch the Baby-friendly Hospital Initiative, to date, practices vary substantially across maternity clinics, and these are neither standardized nor monitored. Currently, there are no Baby Friendly maternity hospitals or clinics in Cyprus and the process of launching a national BFHI accreditation programme is currently in progress. The fragmented implementation of the “10 Steps” and the effect on breastfeeding rates during the first 6 months of life highlight the importance of prioritizing the speedy implementation of the BFHI accreditation scheme in Cyprus to facilitate the protection, support and promotion of breastfeeding. Future research should adopt a holistic approach to assess the determinants of breastfeeding across all levels of the socio-ecological model. By examining individual, interpersonal, community, organizational, policy, and environmental factors, this comprehensive framework will provide a deeper understanding of the multifaceted influences on breastfeeding behaviors.

## Data Availability

The raw data supporting the conclusions of this article will be made available by the authors, without undue reservation.
