# Supplementary material for: Associations between breastfeeding and self-reported experience of the “10 Steps to Successful Breastfeeding”: a cross-sectional and longitudinal study of maternity clinic practices in Cyprus
Source: Front Glob Womens Health. 2024 Dec 16;5:1420670. doi: 10.3389/fgwh.2024.1420670 (PMC11683129; doi:10.3389/fgwh.2024.1420670)
Supplement: Supplementary file 1 [file Table1.docx]

Table 1S: *Definition and Operationalization of the 10 steps as used for the purposes of the study*

| Steps | Step Description/ Components | Step Definition for the estimation of BF and EBF rates | Step Definition for the investigation of the association with BF/EBF initiation and continuation |
| --- | --- | --- | --- |
| Step 3: | Any information given per partum on the following:   - Importance of Skin-to-Skin - Importance of Rooming-in - The risks of giving water, formula or other supplements | **No implementation:** None of the components of Step 3 is implemented   - *Information on the importance of Skin-to-Skin* - *Information on the importance of Rooming-in* - *Information on the risks of giving water, formula or other supplements*   **Partial:** at least one component of Step 3 is implemented  **Full:** all components implemented | **No implementation:** None of the components of Step 3 is implemented  **Partial:** at least one component of Step 3 is implemented  **Full:** all components implemented |
| Step 4: | Mother held the infant within one hour after birth (half an hour)   - Held the infant after birth (within 1 hour) - Skin-to-Skin | **No implementation:** none of the following is implemented:   - “*immediately and within 5 mins* - “*skin to skin’*   **Skin to skin**: “*skin to skin’* is implemented  **“Held the baby within 5 mins after birth”:**  One of the following is implemented: “*immediately”* or *within 5 mins after birth*  **Full:** Both components of Step 4 are implemented | **No implementation:** none of the following is implemented:   - “*immediately and within 5 mins* - “*skin to skin’*   **Partial:** at least one of the components “*Skin to skin”* or “*Holding the baby within 5mins”* is implemented  **Full:** Both components of Step 4 are implemented |
| Step 5: | - Help offered by staff on breastfeeding - Help given on positioning and attaching the baby | **No implementation**: None of the components of step 5 was implemented  **Help offered**: The component *help offered* was implemented  **Practical assistance**: The component practical assistance offered was implemented  **Full:** Both components of Step 5 were implemented: | **No implementation**: None of the components of step 5 was implemented  **Partial:** One of the two components was implemented  **Full:** Both components of Step 5 were implemented |
| Step 7: | Rooming-in | **Implementation:** Step 7 (*Rooming in*) was implemented | **Implementation:** Step 7 (*Rooming in*) was implemented |
| Step 8 | 1. Advice given on the frequency of breastfeeding 2. Advice given on the duration of breastfeeding | **No Implementation:** None of the components of step 8 was implemented  **Any time the baby wants to:** The component ‘*Every time the baby wants to*’ was implemented  **As long as the baby wants to:** The component ‘*As long as the baby* *was wants to*’ was implemented | **No Implementation:** None of the components of step 8 was implemented  **Partial:** one of the two components was implemented  **Full:** Both components of Step 8 were implemented |
| Step 9 | Use of pacifier or other artificial teats (dummies) | **Implementation:** Step 9 (*No use of pacifier or other artificial teats*) was implemented | **Implementation:** Step 9 (*No use of pacifier or other artificial teats*) in was implemented |
| Step 10: | Support provided after discharge | **Implementation:** Step 10 (*guidance provided on how or where to get help after discharge*) in was implemented (Binary variable) | **Implementation:** Step 10 (*guidance provided on how or where to get help after discha*rge) was implemented |
|  | Implementation of the ICBSP | **Implementation:** **ICBSP** (*Leaflets or supplies that promote breast-milk substitutes were not given*) was implemented | **Implementation:** **ICBSP** (*Leaflets or supplies that promote breast-milk substitutes were not given*) was implemented |
