# Supplementary figures and images for: Associations between breastfeeding and self-reported experience of the “10 Steps to Successful Breastfeeding”: a cross-sectional and longitudinal study of maternity clinic practices in Cyprus
Source: Front Glob Womens Health. 2024 Dec 16;5:1420670. doi: 10.3389/fgwh.2024.1420670 (PMC11683129; doi:10.3389/fgwh.2024.1420670)

**S1 Figure:** Participant Flow diagram


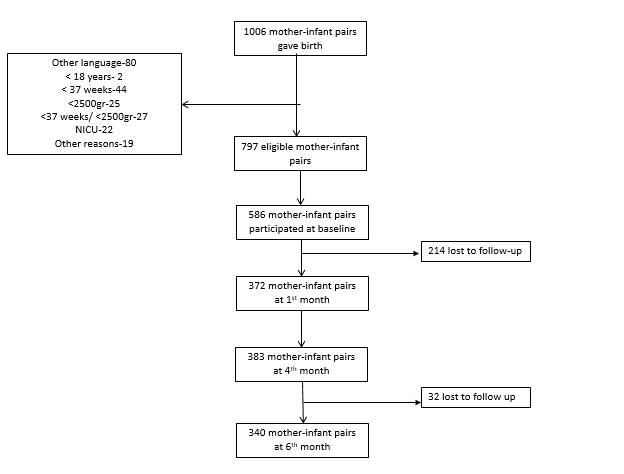

Supplement: Supplementary file 2 [file Table2.docx]

**Figure S1:** Participant Flow diagram


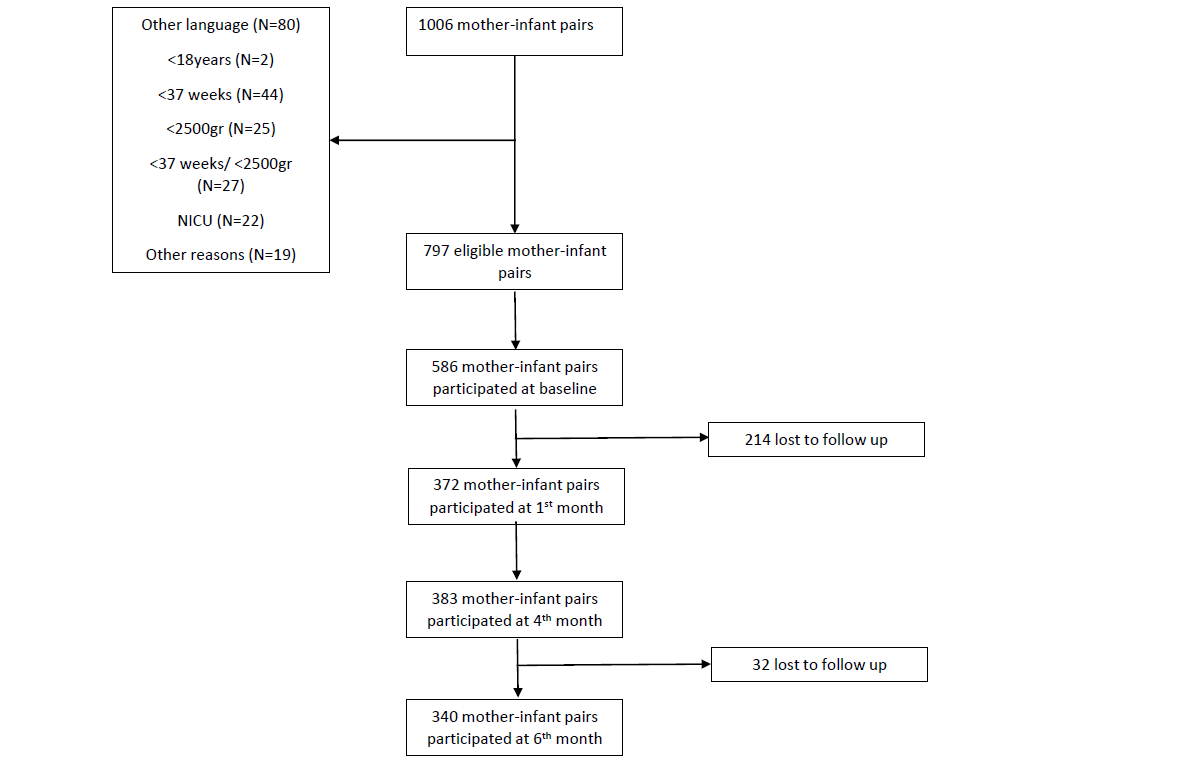

Supplement: Supplementary file 3 [file Datasheet1.docx]
